# Accelerated Aging in HIV/AIDS: Novel Biomarkers of Senescent Human CD8+ T Cells

**DOI:** 10.1371/journal.pone.0064702

**Published:** 2013-05-22

**Authors:** Jennifer P. Chou, Christina M. Ramirez, Jennifer E. Wu, Rita B. Effros

**Affiliations:** 1 Department of Pathology and Laboratory Medicine, University of California Los Angeles, Los Angeles, California, United States of America; 2 Department of Biostatistics, Fielding School of Public Health, University of California Los Angeles, Los Angeles, California, United States of America; 3 Department of Pathology and Laboratory Medicine, UCLA AIDS Institute, David Geffen School of Medicine, University of California Los Angeles, Los Angeles, California, United States of America; University of Modena & Reggio Emilia, Italy

## Abstract

Clinical evaluation of immune reconstitution and health status during HIV-1 infection and anti-retroviral therapy (ART) is largely based on CD4+ T cell counts and viral load, measures that fail to take into account the CD8+ T cell subset, known to show features of accelerated aging in HIV disease. Here, we compare adenosine deaminase (ADA), glucose uptake receptor 1 (GLUT1), and leucine-rich repeat neuronal 3 (*LRRN3*) to CD38 expression and telomerase activity, two strong predictors of HIV disease progression. Our analysis revealed that reduced ADA, telomerase activity and *LRRN3* gene expression were significantly associated with high CD38 and HLA-DR in CD8+ T cells, with % ADA+ cells being the most robust predictor of CD8+ T cell activation. Our results suggest that ADA, *LRRN3* and telomerase activity in CD8+ T cells may serve as novel, clinically relevant biomarkers of immune status in HIV-1 infection, specifically by demonstrating the degree to which CD8+ T cells have progressed to the end stage of replicative senescence. Since chronological aging itself leads to the accumulation of senescent CD8+ T cells, the prolonged survival and resultant increased age of the HIV+ population may synergize with the chronic immune activation to exacerbate both immune decline and age-associated pathologies. The identification and future validation of these new biomarkers may lead to fresh immune-based HIV treatments.

## Introduction

Clinical evaluation of immune reconstitution and health status during HIV-1 infection and anti-retroviral therapy (ART) is largely based on CD4+ T cell counts and viral load, measures that provide a somewhat incomplete picture of the true functional status of the immune system. Specifically, these parameters fail to consider the state of CD8+ T cells, the subset primarily responsible for controlling the infection through lysis of HIV-1-infected cells, release of perforin and granzyme, production of IFN-γ and secretion of soluble factors that suppress HIV-1 replication [1], [2]. Since HIV disease involves accelerated immunological aging [3], research that focuses on a cell population that undergoes significant changes during chronological aging may provide novel insights into HIV pathogenesis. Indeed, both HIV disease and aging involve the accumulation of a population of dysfunctional CD8+ T cells with characteristics of cellular (replicative) senescence, an end stage characterized by irreversible cell cycle arrest, multiple genetic and functional changes, and shortened telomeres. The clinical relevance of this cell population is underscored by several observations. First, CD8+ T cells with several signature features of replicative senescence, including reduced anti-HIV effector functions, permanent suppression of CD28 gene expression, critically short telomeres, and loss of the ability to upregulate telomerase, are significantly increased in HIV-1-infected persons, even in those on ART [4]. Moreover, the abundance of CD8+CD28- T cells early in the infection is actually predictive of the subsequent rate of progression to AIDS [5]. Given the prolonged survival and “graying” of the HIV-infected population, it is possible that the CD8+ T cell defects due to the infection may synergize with similar defects associated with aging, further highlighting the critical need for more precise characterization of this subset.

Based on the significant increase in CD8+ T cells with markers of senescence in HIV-infected persons, the current study focused on several novel markers of replicative senescence (ADA, GLUT 1 and LRRN3) to determine their relationship to the elevated CD38 expression that has been significantly linked to adverse outcomes in HIV disease [6]. CD38 is a multifunctional ectoenzyme involved in the regulation of intracellular calcium. Loss of CD38 function is associated with impaired immune responses and metabolic disturbances, while increased CD38 surface expression is a marker of immune activation that has been linked not only to HIV, but also to B cell malignancies, solid tumors and type 2 diabetes [7].

Among the markers to be tested, adenosine deaminase (ADA), an ectoenzyme present on T cells, seemed particularly relevant to HIV disease, since it functions to convert immunosuppressive adenosine to inosine. Adenosine not only inhibits multiple T cell functions, including IL-2 production [8], [9], [10], but also accelerates the generation of senescent T cells and loss of CD28 in cell culture [11]. ADA also enhances CD28 costimulation by amplifying the immunological signaling through its binding to CD26 on antigen-presenting cells. (Interestingly, in HIV-infected persons, ADA binding to CD26 is inhibited by HIV glycoproteins and viral particles [12], [13]). Importantly, our recent studies demonstrated that CD8+CD28+ T lymphocytes lacking ADA surface expression (i.e.,ecto-ADA) have significantly less telomerase activity than those that do express ADA, and that ADA surface receptor expression continuously declines during the progression of T cells to senescence in cell culture [11]. Expression of the GLUT 1 receptor, which regulates T cell glucose uptake, is modulated by CD28 [14], [15]. Like ADA, its expression declines in parallel with the transition of chronically activated CD8+ T cells to the end stage of senescence in culture, and the decline coincides with reduced gene expression of key enzymes involved in gluconeogenesis and glycolysis. The third novel biomarker selected for analysis with respect to CD8+ T cells in HIV disease is the Leucine-Rich Repeat Neuronal 3 gene (*LRRN3*), the expression of which declines in senescent CD8+ T cell cultures, and in *ex vivo* purified senescent CD8+ T cells from HIV-infected persons (unpublished data). Importantly, causal network analysis [16] of gene expression data from multiple independent aging studies suggests that LRRN3 lies upstream of CD28 (Horvath,S., Effros, R.B., manuscript in preparation).

Here, we present our initial studies on T cells from 48 HIV-infected men, and show that ADA and LRRN3, as well as telomerase activity, strongly correlate with CD8+ T cell activation status as measured by CD38 and HLA-DR surface expression. Our analysis revealed that among the markers tested, ADA expression showed the strongest association, independent of a variety of confounding factors. There was no association detected between activation markers and the GLUT 1 glucose transporter.

## Materials and Methods

### Ethics Statement

All study participants from the cross-sectional study were recruited from the Los Angeles area. This study was approved by the University of California, Los Angeles Medical Institutional Review Board and each participant provided written, informed consent per the approved protocol.

### Human Subjects

HIV+ males, who are participants in the UCLA Multicenter AIDS Cohort Study (MACS) provided peripheral blood samples during their routine MACS Clinic visit. At the time of the visit, clinical information was obtained and confirmed by medical record review. Data on HIV-1 serostatus, absolute CD4+ T-cell counts, and plasma HIV viral load were collected. All participants are on combination antiretroviral therapy. Table 1 summarizes the clinical data of the study participants.

**Table 1:** Age and Clinical Features of Study Participants (N = 48).

Demographic Characteristics	Mean	Range
**Mean age (SD)**	57.5 (6.4)	41–67 years
**Duration of Infection (SD)**	22.9 (5.3)	7.3–25.6 years
**AIDS diagnosis (% total)**	11 (23%)	
**%CD4 positive (SD)**	31.4% (9.1)	11–51%
**Viral Load in copies/mL**	10*	Undetectable (10) -9580
**Other diagnostic diseases**	# cases (% of total)	
Wasting Syndrome	4 (8.3%)	
Pneumocystis pnemonia	2 (4.2%)	
Crytosporidiosis	1 (2.1%)	
Pulmonary tuberculosis	1 (2.1%)	
Non-hodgkin's lymphoma	1 (2.1%)	
Candida esophagitis	1 (2.1%)	
Multiple AIDS diagnosis	1 (2.1%)	

All participants are on combination antiretroviral therapy.

*The median is provided because there were a few outliers with very high viral load.

### Cell isolation and activation

Fresh peripheral blood was acquired by venipuncture. Following Ficoll-Hypaque gradient centrifugation, the PBMC layer was carefully removed and washed twice in complete RPMI 1640 (10% FBS, 10 mM HEPES, 2 mM glutamine, 50 IU/ml penicillin/streptomycin). For LRRN3 studies, CD8+ T cells were isolated immediately after Ficoll-Hypaque centrifugation, using the EasySep CD8 enrichment kit (Stemcell Technologies, Vancouver, British Columbia, Canada). For the telomerase activity assay, cell activation was necessary, since our previous studies indicated that telomerase is undetectable in *ex vivo* isolated CD8+ T cells from HIV-infected persons. Accordingly, PBMCs were exposed for 72 hours to T cell activation microbeads (anti-CD2/3/28; Miltenyi Biotec, Auburn, CA), with 10 µl microbead mixture added for every 1×10^6^ cells, and CD3+ and CD8+ T cells were isolated using EasySep human T cell or CD8+ enrichment kits (Stemcell Technologies, Vancouver, British Columbia, Canada). For flow cytometry experiments, PBMCs were cultured for 72 hours with irradiated (8000 rad) allogeneic transformed B lymphoblastoid cells in complete RPMI 1640 supplemented with recombinant IL-2 (10 U/ml), as previously described [17]. For all cultures, viable cell concentration was routinely >90%, as determined by trypan blue exclusion. Purity of the CD8+ T cells was verified by flow cytometry and was routinely >95%. For these studies, rather than separating only the HIV-1-specific CD8+ T cells, we isolated total CD8+ T cells, because that allowed us to capture information on CD8+ T cells of multiple specificities, which are putatively being chronically stimulated by the inflammatory milieu, and/or by other latent viruses, such as EBV and CMV.

### Flow cytometry

Surface expression of CD8, CD3, CD28, GLUT1, and ADA was examined by immunostaining and flow cytometry. Cells were incubated with FITC-conjugated anti-GLUT1 (R&D Systems, Minneapolis, MN), PE-conjugated anti-CD28, APC-conjugated anti-CD3, and PerCP-conjugated anti-CD8 (BD Biosciences, San Jose, CA), or FITC-conjugated anti-CD3, PE-conjugated anti-CD28, anti-ADA biotinylated (Abcam, Cambridge, MA) with strepavidin-PerCP, and APC-conjugated anti-CD3 (BD Biosciences, San Jose, CA) mAbs at 4°C for 20 min, washed, and fixed in PBS containing 1% paraformaldehyde. Staining with primary and secondary mAbs alone was included as controls for ADA, to ensure exclusion of non-specific or background fluorescence. Parallel samples were incubated with Ig isotype control Abs or secondary Abs (BD Biosciences). All samples were analyzed on a FACS Calibur flow cytometer (BD Biosciences). Fluorescence data from at least 25,000 cells were acquired. The lymphocyte population was gated on, and within that population, CD3, CD28, CD8, GLUT1 and ADA positive fluorescence were assessed, taking into account non-specific isotype staining and appropriate primary and secondary control stains. Analysis of data was performed using CellQuest Pro (BD Biosciences). We included samples from two healthy subjects in our flow cytometry analysis, and, as expected they showed no staining with the activation markers, and positive staining for GLUT 1 and ADA.

### Telomerase activity measurements

Telomerase activity was determined using a modified version of the Telomerase Repeat Amplification Protocol (TRAP), as previously described [18], Briefly, for each sample 1×10^6^ CD3+ or CD3+CD8+ cells were pelleted and washed twice with PBS. Cell pellets were lysed in 100 uL of M-PER Mammalian Protein Extraction Reagent (Pierce, Rockford IL) and incubated on ice for 1 hour. To control for slight inter-sample cell number variance, samples were normalized according to nucleic acid concentration, which was determined using the Quant-iT Ribogreen RNA Assay Kit (Molecular Probes). The endogenous telomerase present in the cell extract adds telomeric repeats to the telomerase substrate (TS), a nontelomeric oligonucleotide. The extension products are then amplified several-fold by the polymerase chain reaction (PCR) carried out by *Taq polymerase* using a Cy-5-labeled forward primer (known as TS: 5′-/5Cy5/AATCCGTCGACGCAGAGTT) as a substrate for telomerase-mediated addition of TTAGGG repeats, and an anchored reverse primer (ACX 5′-GCGCCGCTTACCCTTACCCTTACCCTAACC-3′). To measure the telomerase activity, each sample was mixed with 20 ul of Bromothenol Blue loading dye, and 35 ul of sample + dye and was loaded and run using 10% non-denaturing PAGE in 1×TBE buffer. Gels were run at approximately 300 V for 80 min, and each sample was evaluated at least two times on separate gels. Gels were scanned on a STORM 865 (GE Healthcare, Piscataway, NJ) and quantified using the software ImageQuant 5.2, which integrates signal intensity over the telomere length distribution on the gel as a function of molecular weight (GE Healthcare, Piscataway, NJ).

### Real-Time Quantitative Polymerase Chain Reaction of LRRN3

RNA was isolated using the Qiagen RNAeasy kit (Qiagen, Valencia, CA), and quantified using the Quant-iT Ribogreen RNA Assay Kit (Molecular Probes). cDNA was synthesized with the iScript cDNA synthesis kit (Bio-Rad) using 300 ng RNA. Real-time quantitative polymerase chain reaction assays were performed using the SsoFast EvaGreen dye (Bio-Rad) and samples run on CFX96 (Bio-Rad), using 36B4 as an internal control. The sequences were designed with the aid of Primer 3 software and synthesized by Integrated DNA Technologies, Inc., Coralville, IA. LRRN3: sense TCCTTACCTAGACAATGAGAAGAGCAATC, antisense CCACCAACCACCACCAGCAC; 36B4: sense CAGGCT-AACTACTCGCTCAACACAG; antisense TGTCCCGTTTGGTCATCTGGTAATC.

### Statistical Methods

Correlation was assessed by Pearson correlation. Linear regression was performed controlling for the following confounders: log viremia, CD4+ T cell count, an indicator whether the patient has been diagnosed with an AIDS-defining opportunistic infection, and age. Viral load measures were log 10 transformed prior to analysis. Analyses were performed in SAS version 9.2 (Cary, NC) and R for Mac version 2.15.

## Results

### ADA expression is inversely correlated with CD8+ T cell activation markers

**Figure 1:**
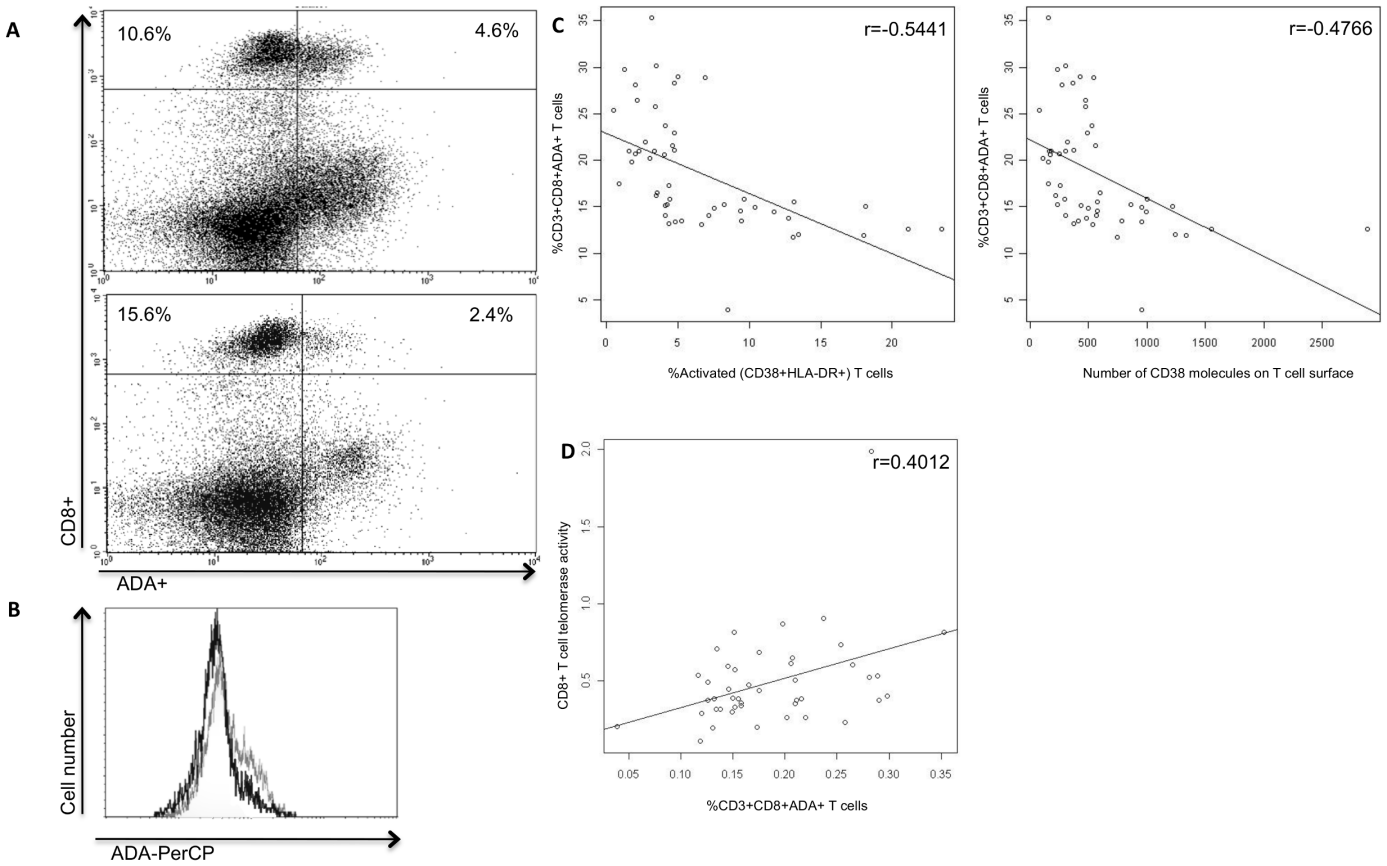
Low ecto-ADA expression correlates with high levels of activation markers. (a) Representative flow cytometry data of surface staining for ecto-ADA and CD8 molecules on T cells 4 days after stimulation with an allogeneic transformed B-cell line. Comparison between %ADA+ within the CD8+ T cell subset from HIV+ donors with low (top) and high activation (bottom) levels, defined by activation marker CD38 (2.1% and 18.2%, respectively). CD8+ T cells with low %CD38 had greater ecto-ADA expression (30.2%), as compared to those with high %CD38, who showed lower ADA (13.3%). (b) Representative histogram comparing the same donors as in panel (a). The dark lines represent the donor with high activation (18.2%), compared to the donor with low activation (2.1%). (c) Statistical analyses indicating a strong negative relationship between ecto-ADA expression and percentages of CD38+ and HLA-DR+ (r = −0.5441, p<0.0001), as well as number of CD38 molecules (r = −0.4766, p = 0.0004). (d) Significant positive correlation between CD8+ADA+ and CD8+ telomerase activity (r = 0.4012, p<0.0069).

PBMCs were isolated from 48 HIV-1+ individuals and the extracellular phenotypes of CD8+ T cells within these populations were investigated by flow cytometry. Using the Pearson Correlation model, we found that persons with the lowest percentage of ADA on total CD8+ T cells had the highest number of surface CD38 and HLA-DR molecules, as well as the highest percentage of CD8+ T cells that were positive for both activation markers (fig. 1a-1c). Furthermore, consistent with our previous *in vitro* studies on T cells from HIV-infected donors, which documented the highest telomerase activity in those CD8+CD28+ T cells that also expressed ADA [11], the current analysis shows that CD8+ T cell telomerase activity was positively correlated with the percentage of CD8+ T cells that were ADA+ (fig. 1d, r = 0.4012, p = 0.0069).

In addition, using regression analysis and controlling for such confounding factors as log viremia and low CD4+ T cell counts, the data indicate that ADA expression on CD8+ T cells could explain nearly 53% of the variance in the levels of CD38 and HLA-DR activation observed (p<0.0001). These activation markers are commonly used in clinical settings to assess immune status and the manifestation of AIDS. We conclude from our results that loss of ecto-ADA on CD8+ T cells may reflect a more senescent and dysfunctional phenotype, and that HIV-infected persons with low ADA expression on their CD8+ T cells may undergo a more rapid progression to AIDS. Therefore, inclusion of surface ADA expression analysis in the assessment of immune status during chronic HIV-1 infection may provide a new, biologically meaningful clinical metric, particularly with respect to its role in enhancing telomerase activity.

### Higher *LRRN3* gene expression correlates with a less senescent T cell phenotype

**Figure 2:**
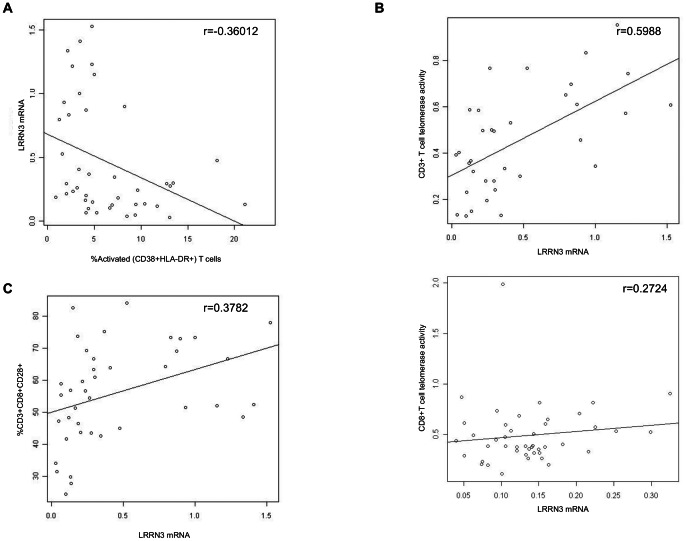
LRRN3 mRNA expression is associated with increased expression of CD38+ and HLA-DR+. (a) *Ex vivo* CD8+ T cells from HIV+ donors were isolated using RosetteSep CD8+ T cell enrichment cocktail from whole blood. *LRRN3* transcription in CD8+ T cells was compared for HIV+ donors with high and low levels of activation, defined by CD38 and HLA-DR. 36B4 was used as the housekeeping gene. There was a strong negative relationship between *LRRN3* mRNA and percentages of CD38+ and HLA-DR+ cells (r = −0.36012, p = 0.0192). (b) Positive correlation between *LRRN3* mRNA and CD3+ telomerase activity (r = 0.5988, p<0.0001) and CD8+ telomerase activity (r = 0.2724, p = 0.11). (c) Positive relationship between *LRRN3* mRNA and %CD28+ within the CD8+ T cell subset (r = 0.3782, p = 0.0161), suggesting a potential modulatory role of *LRRN3* on CD28 surface expression.

Gene expression of *LRRN3* was inversely correlated with the percentage of CD38+HLA-DR+CD8+ T cells (fig. 2a, r = −0.36012, p = 0.0192). Furthermore, *LRRN3* expression was strongly positively correlated with telomerase activity of T cells (fig. 2b, r = 0.5988 p = 0.0002), underscoring the potential upstream regulatory role of *LRRN3* in T cell functionality. In that regard, our data show that the level of *LRRN3* mRNA was also positively correlated (fig. 2c, r = 0.3782, p = 0.0161) with the percentage of CD8+ that express CD28, a key costimulatory receptor required for optimal T cell activation, telomerase upregulation, signal transduction and function [19], [20], [21]. Since irreversible loss of CD28 gene expression is one of the key characteristics of senescence, this positive association highlights a possible immunological interaction between these genes, and suggests that expression of *LRRN3*, like *CD28*, is important for the prevention of acquiring features of senescence. Thus, our data support the hypothesis that sustained expression of *LRRN3* may function to maintain normal T cell function during chronic antigenic stimulation, similar to our observations regarding telomerase [22].

### High CD38 expression was strongly associated with reduced telomerase activity

**Figure 3:**
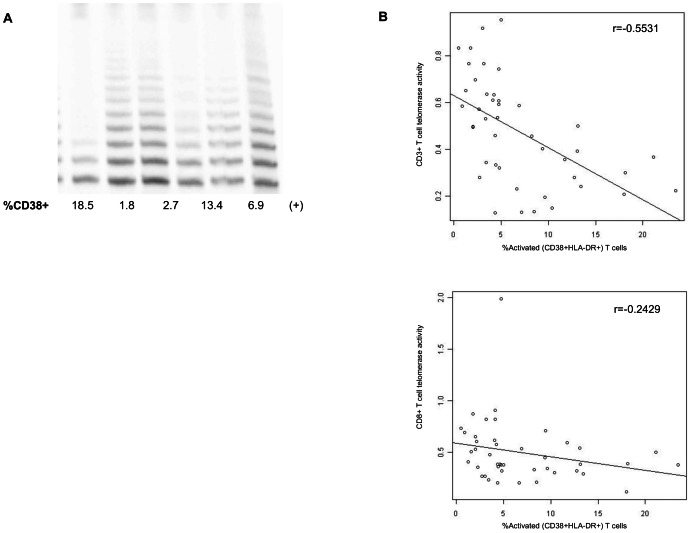
Telomerase activity is inversely correlated with activation biomarkers in T cells. (a) Representative telomeric repeat amplification protocol (“TRAP”) gel of CD8+ T cells from individual HIV+ donors tested 4 days after activation with anti-CD2/CD3/CD28 microbeads. Lane 1 (+) shows telomerase activity of the Jurkat T cell line. All telomerase activity values from each donor were normalized relative to this positive control. The CD8+ T cell telomerase activity was compared to the percent CD38+. Low %CD38 CD8+ T cells (lanes 2 and 3, with 1.8% and 2.7% CD38+, respectively) have significantly more telomerase activity, indicated visually by the darker bands, than CD8+ T cells with high %CD38+ (lane 1, with 18.5% CD38+). (b) Telomerase activity of CD3+ T cells and the CD8+ T cell subset from HIV+ donors was compared to the %CD38+HLADR+. Statistical analyses showed a strong negative relationship between the %CD38+DR+ cells and telomerase activity of both CD3+ (r = −0.5531, p = 0.0001) and a trend with CD8+ T cells (r = −0.2429, p = 0.1149).

Our analysis of telomerase activity accords with studies by Lichterfeld et al. [23], showing that CD8+ T cells from HIV-1 controllers have long telomeres and high levels of constitutive telomerase activity, while the opposite phenotype characterizes T cells from progressors. Indeed, we observed that T cells from individuals with a high percentage of CD8+ T cells that were strongly positive for CD38 and HLA-DR had significantly lower telomerase activity than T cells with low levels of these activation markers. Fig. 3a is a representative TRAP gel showing CD8+ T cell telomerase activity from two donors, one with high, and one with low percentages of CD8+ T cells that were CD38+. Our collective data set documents a strong negative relationship between the proportion of activated (CD38+HLA−DR+) T cells and telomerase activity of total (CD3+) T cells (fig. 3c, r = −0.5531, p = 0.0001). Moreover, as noted above, there was a strong association between ADA expression and CD3+ T cell telomerase activity (not shown, r = 0.6511; p<0.0001), further underscoring the importance of ADA in modulating telomerase, as proposed by Parish et al. [11]. Reduced telomerase activity is a well-established marker of senescence in aging and HIV-1 infection both *in vitro* and *in vivo* [23], [24], [25]. Thus, the reduced telomerase activity observed in the HIV-1-infected MACS donors with high percentages of CD38 and HLA-DR within our sampling group supports our overarching hypothesis that chronic activation is a strong driver of the progression of CD8+ T cells along the trajectory to replicative senescence. These data add to the accumulating evidence that the process of replicative senescence, which affects multiple facets of human aging, also contributes to the overall adaptive immune system dysfunction during HIV-1 infection.

## Discussion

The major finding of the current study relates to ADA, which when complexed to CD26 on antigen-presenting cells, constitutes a key costimulatory component of the immunological synapse. Our analysis provides the first demonstration that ADA is significantly reduced in CD8+ T cells from those HIV-1-infected individuals with high CD38 expression, a biomarker of immune activation. This observation is consistent with extensive data on loss of ADA expression caused by chronic activation of CD8+ T cells in cell culture [11]. Using regression analysis, ADA positivity was also found to serve as the strongest variable among our biomarkers that accounts for more than half of variation of expression of CD38+HLA-DR+, markers frequently used in clinical settings to evaluate HIV disease progression. In addition, since ADA is essential for optimal telomerase activity in CD8+ T lymphocytes [11], the results of this study, indicating the inverse relationship between CD38 and T cell telomerase activity, may support the upstream stimulatory function of ADA on telomerase activity. This observation has important *in vivo* consequences, since T cells lacking ADA are vulnerable to adenosine, an immunosuppressive factor produced by certain Tregs and present in the microenvironment of many tumors [26]. Moreover, exposure of CD8+ T cells to adenosine in cell culture accelerates many of the changes associated with senescence, such as reduced proliferative potential, diminished IL-2 message, and early loss of both CD28 expression and telomerase activity [11].

Our study also demonstrates strong associations between telomerase activity, *LRRN3* gene expression and CD38 levels, and may therefore establish a novel panel of parameters that can be used in future clinical evaluation of our hypothesis. Together, the data provide a more definitive picture of the dysfunctional CD8+ T cell compartment in HIV-infected persons. Indeed, further refining of the genetic and molecular signature of senescent CD8+ T cells in the HIV-1-infected population may be beneficial in assisting clinicians to strategize the course and intensity of ART treatment for individual patients. Interestingly, although GLUT 1 expression and glucose uptake in T cells are both modulated by CD28, our analysis failed to show any association between GLUT 1 expression and CD8+ T cell activation.

The accumulation of senescent CD8+ T cells affects not only immune function, but may also impact other physiological systems. Indeed, a recent study by Kaplan et al. [27] showed that frequencies of both highly activated (i.e., CD8+CD38+HLA-DR+) and senescent (i.e., CD8+CD28−CD57+) T cells in HIV-1-infected women were associated with increased prevalence of carotid artery lesions. It was concluded that HIV-associated T cell changes, characterized by specific senescence and activation markers, are associated with subclinical carotid artery abnormalities, which are observed even among those patients achieving viral suppression with effective ART [27]. Collectively, those results, coupled with our own findings, provide compelling evidence for the importance of incorporating CD*+ T cell senescence-related biomarkers in both the clinical evaluation of immune status and in determining risk factors for age-related pathologies that are occurring with increasing frequency in the context of HIV disease.

In sum, these initial observations on novel molecular and genetic changes occurring as CD8+ T cells progress toward the end stage of senescence in HIV-1 infected persons highlight the need for more comprehensive *in vitro* proof-of-principle and functional analyses of the causal role of these markers in the generation of senescent CD8+ T cells. Gene therapy with viral vectors encoding ADA, LRRN3 and/or hTERT, the catalytic subunit of human telomerase, as well as testing small molecules that upregulate enzymatic or transcriptional activity of these genes may lead to further elucidation of critical new pathways involved in the process of cellular senescence. Current research in our lab is addressing these issues, with the goal of developing fresh therapeutic approaches to rejuvenating immune function of CD8+ T cells, in order to enhance the health span of the aging HIV-1-infected population.
